# Deciphering the Effective Constituents and Mechanisms of *Portulaca oleracea* L. for Treating NASH *via* Integrating Bioinformatics Analysis and Experimental Pharmacology

**DOI:** 10.3389/fphar.2021.818227

**Published:** 2022-01-19

**Authors:** Xiaoli He, Yiren Hu, Wei Liu, Guanghao Zhu, Ruoxi Zhang, Jiawen You, Yanting Shao, Yunhao Li, Zeng Zhang, Jingang Cui, Yanming He, Guangbo Ge, Hongjie Yang

**Affiliations:** ^1^ Department of Endocrinology, Research Laboratory of Pharmacy, Center of Experimental Animals, Clinical Research Institute of Integrative Medicine, Yueyang Hospital of Integrated Traditional Chinese and Western Medicine, Shanghai University of Traditional Chinese Medicine, Shanghai, China; ^2^ Key Laboratory of Liver and Kidney Diseases (Ministry of Education), Institute of Liver Diseases, Shuguang Hospital Affiliated to Shanghai University of Traditional Chinese Medicine, Shanghai, China; ^3^ Shanghai Frontiers Science Center of TCM Chemical Biology, Institute of Interdisciplinary Integrative Medicine Research, Shanghai University of Traditional Chinese Medicine, Shanghai, China

**Keywords:** *Portulaca oleracea* L., non-alcoholic steatohepatitis, prostaglandin-endoperoxide synthase 2, myricetin, hepatic steatosis

## Abstract

Nonalcoholic steatohepatitis (NASH) is a highly prevalent metabolic disorder. Currently, there are no effective pharmacotherapeutic options for preventing and treating NASH. *Portulaca oleracea* L. (POL) is an edible herb that has been used for preventing and treating some metabolic disorders in China, but the bioactive constituents in POL and the related mechanisms for treating NASH are still unclear. Here, a comprehensive research strategy was used to identify the core genes and the key constituents in POL for treating NASH, *via* integrating bioinformatics analysis and experimental pharmacology both *in vitro* and *in vivo*. The phenotypes and mechanisms of POL were carefully investigated by performing a set of *in vivo* and *in vitro* experiments. Bioinformatics analysis suggested that prostaglandin-endoperoxide synthase 2 (PTGS2) was the core target and myricetin (Myr) was the key constituent in POL for treating NASH. In NASH mice model induced by methionine choline deficiency diet, POL significantly alleviated hepatic steatosis and liver injury. In free fatty acids-induced hepatocytes, POL and Myr significantly down-regulated the expression of PTGS2, decreased the number of lipid droplets, and regulated the mRNA expression of lipid synthesis and homeostasis genes, including *FASN*, *CPT1a*, *SERBP1c*, *ACC1*, and *SCD1*. In lipopolysaccharide-induced macrophages, POL and Myr significantly reduced the expression of PTGS2 and blocked the secretion of inflammatory mediators TNF-α, IL-6, and IL-1β. Further investigations demonstrate that Myr acts as both suppressor and inhibitor of PTGS2. Collectively, POL and its major component Myr can ameliorate NASH *via* down-regulating and inhibiting PTGS2, suggesting that POL and Myr can be developed as novel medicines for treating NASH.

## Introduction

Non-alcoholic fatty liver disease (NAFLD) is a systemic metabolic disorder. Excessive hepatic accumulation of lipids and chronic inflammation are the main histopathological features among patients with NAFLD. When simple non-alcoholic fatty liver (NAFL) evolves into non-alcoholic steatohepatitis (NASH), it will develop severe hepatic complications, such as liver fibrosis, cirrhosis, or even hepatocyte carcinoma (Younossi et al., 2019). NASH is closely related to several metabolic diseases far beyond the liver, such as obesity, hyperglycemia, hyperlipidemia, and hyperuricemia. NASH is also an independent risk factor of type 2 diabetes mellitus, hypertension, atherosclerosis, and cardiovascular disease (Lonardo et al., 2018). The prevalence of NAFLD is continuously increasing worldwide given the vigorous increase in the number of obese patients. Large-scale epidemiologic data reported approximately 2 billion patients with NAFLD globally and more than 1.1 billion in Asia (Younossi et al., 2016). The pathogenesis of NASH is complex and remains unclear. With further research in recent years, the understanding of the mechanism underlying the development of NASH changes from two-hit hypothesis to multiple-hit hypothesis. Comprehensive pathological hits including insulin resistance, hormones secreted from the adipose tissue, nutritional factors, gut microbiota, and genetic and epigenetic factors play a common important role from the onset to evolution of NASH (Friedman et al., 2018). Thus far, no effective pharmacotherapy is available for treating NASH due to its complex pathogenesis.

*Portulaca oleracea* L. (POL) is an annual herb widely distributed in subtropical and tropical areas. It is also an edible vegetable frequently used in cooking soup and salad in Mediterranean diet which is benefit to alleviate NAFLD (Zhou et al., 2015). It is traditionally used to treat hyperlipidemia, hyperglycemia, obesity, and other metabolic disorders (Zidan et al., 2014; Lee et al., 2020; Darvish et al., 2021; Sargin et al., 2021). POL seeds have beneficial effects on regulating serum glucose and lipids, and increasing insulin sensitivity in patients with NAFLD (Gheflati et al., 2019). However, whether POL can alleviate NASH and its possible mechanisms are still unclear. POL are rich of flavonoids including myricetin, quercetin, kaempferol, luteolin, apigenin, etc. which have extensive pharmacological effects (Nemzer et al., 2020). Recent studies reported that myricetin could treat NASH by modulating macrophage polarization and mitigating liver inflammation and fibrosis *in vivo* (Yao et al., 2020). And it could decreases hepatic lipid synthesis and inflammation by modulating gut microbiota, regulating Nrf2 and peroxisome proliferator activated receptor (PPAR) signaling pathways (Chang et al., 2012; Xia et al., 2016; Sun et al., 2021). In this study, we try to evaluate the pharmacological effect of POL in treating NASH and explore its main active components.

The pharmacological mechanism of Chinese medicines are always complex and hardly to elucidate, owing to the larger number of components in CMs. Network pharmacology analysis is widely used to analyze the active components and possible targets of natural plant medicine and formulas given the advances in data mining and bioinformatics analysis (Zhao et al., 2021; Zhou et al., 2021). By the integrated methods of abundant database sources and multiple statistical algorithms, network pharmacology can systematically observe and evaluate the synergy of multiple components, targets, and mechanisms of diseases (Kibble et al., 2015; Yang et al., 2020). In the present study, a network pharmacology analysis was employed to discover the active components of POL and their possible targets in treating NASH. Ultra high performance liquid chromatography-Q exactive hybrid quadrupole orbitrap high-resolution accurate mass spectrometric (UHPLC-Q-Orbitrap HRMS), and verification experiments were conducted to determine the efficacy of active components and their mechanisms in treating NASH.

## Materials and Methods

### Experimental Animals and Diets

Thirty-two male C57BL/6 mice (6 weeks old, weighing 20–22 g) were purchased from Jihui (Jihui Experimental Animal Breeding Co., Ltd, Shanghai, CHN) and adaptively fed for 1 week in the Animal Experimental Center of Yueyang Hospital Affiliated to Shanghai University of TCM (License No. SYXK 2018-0040). The mice were randomly assigned to the methionine- and choline-sufficient diet (MCS) group (*n* = 8) and the methionine- and choline-deficient diet (MCD) group (*n* = 24), which were fed with MSC or MCD diet, respectively, for 6 weeks. MCD diet containing 16% kcal protein, 21% kcal fat, and 62% kcal carbohydrate was used to induce NASH mouse model as previously described (Mridha et al., 2017). MCD (Lot No. A02082002B) and MCS (Lot No. A02082003B) diets were purchased from Research Diets (Research Diets Inc., NJ, United States).

### Reagents and Drugs

Fetal bovine serum (FBS), Dulbecco’s modified Eagle Medium (DMEM), penicillin–streptomycin solution, 0.25% trypsin, and phosphate buffer saline (PBS) were purchased from Gibco (Thermo Fisher Scientific Inc., MA, United States). Insulin, lipopolysaccharide (LPS), bovine serum albumin (BSA), dexamethasone, and enzyme-linked immunosorbent assay (ELISA) kits (IL-6, TNF-α, IL-1β) were obtained from Beyotime (Beyotime Biotechnology Inc., Shanghai, CHN). Cell Counting Kit-8 (CCK8) was acquired from Dojindo (Dojindo Laboratories Inc., Kumamoto, JPN). Triglyceride (TG), total cholesterol (TC), alanine aminotransferase (ALT), and aspartate aminotransferase (AST) test kits and oil red O staining kit were provided by Jiancheng (Jiancheng Bioengineering Institute, Nanjing, CHN). Dimethyl sulfoxide (DMSO), oleic acid (OA), palmitic acid (PA), paraformaldehyde, Triton-X 100, and isopropanol were supplied by Sinopharm (Sinopharm Group Co. Ltd, Shanghai, CHN).

Quercetin (BP1187-20 mg, purity ≥98%), myricetin (BP0970-20 mg, purity ≥98%), luteolin (BP0896-20 mg, purity ≥98%), kaempferol (BP0820-20 mg, purity ≥98%), and apigenin (BP0177-20 mg, purity ≥98%) were purchased from Purifa (Purifa Biotechnology Inc., Chengdu, CHN). Plant sample was purchased from Xinxing (Xinxing Chinese Herbal Pieces Co. Ltd., Bozhou, CHN). Then, the plant was identified and authenticated from the Research Laboratory of Pharmacy (Yueyang Hospital of Integrated Traditional Chinese and Western Medicine, Shanghai University of Traditional Chinese Medicine, Shanghai, CHN); a voucher specimen (No. YYZY-210512) has been deposited in herbarium of the same department. Pioglitazone hydrochloride (B21435-100 mg, purity ≥98%) was purchased from Yuanye Biotechnology Co., Ltd. (Shanghai, CHN).

### Different Expression Genes Between the Healths and the Patients With NASH

The high-throughput sequencing data of liver tissues from patients with NASH and healthy people (GSE135251) were obtained from GEO DataSets. ENSG ID was convert into gene symbols by using Uniprot and Ensembl databases. Differentially expressed genes (DEGs) were analyzed by LIMMA package on R software (Version 4.0.3). The absolute value of log_2_ fold change (|logFC|) was set at > 1.5, while the adjusted *p* value (adj.P) was set at < 0.05. The volcano and heatmap plots of DEGs were graphed using R software.

### Screening of Components and Targets of POL in Treating NASH

The components and predicted targets of POL were collected from TCMSP, BATMAN, and TCMID databases. Drugbank and Pubchem were used to search the name and structure of each compound. Uniprot and GeneCards were used to search the names of predicted targets. The predicted targets of POL and DEGs from GSE135251 (N&P) were intersected by Venn diagram.

### Bioinformatics Analysis and Screening of High-Potential Targets

The Gene Ontology (GO) enrichment analysis of N&P, which included the biological process (BP), molecular function (MF), and cellular component (CC) categories, was conducted by R software. The Kyoto Encyclopedia of Genes and Genomes (KEGG) pathway analysis of N&P was conducted using R software. Significant genes or pathways with *p*-value < 0.05 were selected. The bubble and histogram plots of GO and KEGG enrichment were graphed by R software. Protein–protein interaction, component–protein interaction, protein–pathway interaction, and topological analyses of N&P were conducted by Cytoscape software (National Institute of General Medical Sciences, MD, United States).

### Preparation of the Ethanol Extracts of POL

Coarse particles of POL seeds (1000 g) were immersed with five fold volume of 95% ethanol (v/v) for 24 h, extruded, and infiltrated by a filter membrane. The last process was conducted twice. The infiltrated solution of POL was combined and concentrated at 60°C to eliminate ethanol by rotary evaporation. The sticky cream was diluted by deionized water, frozen, and dried at −80°C by a vacuum freeze dryer to obtain the ethanol extract powder of POL.

### Determination of the Contents of Five Flavonoids in POL by UHPLC-Q-Orbitrap HRMS

The contents of five flavonoids in POL were analyzed by using UHPLC-Q-Orbitrap HRMS (Thermo Fisher Scientific Inc., Grand Island, NY, United States). The UHPLC was Thermo Scientific Dionex Ultimate 3000 and controlled by Chromeleon 7.2 Software. The cooling autosampler was set at 10°C and protected from light, and the column heater was set at 45°C. A Waters ACQUITY UPLC BEH C_18_ column (2.1 × 100 mm, 1.7 μm) was employed. The mobile phase consisted of A (methanol) and B (0.1% formic acid) at a flow rate of 0.3 ml min^−1^ and eluted with gradient elution: 0–2.0 min (4% A), 2.0–6.0 min (4–12% A), 6.0–38.0 min (12–70% A), 38.0–38.5 min (70% A), 38.5–39.0 min (70–95% A), 39.0–43.0 min (95% A), 43.0–45.0 min (4% A). The injection volume was 2 μL. The mass spectrometer Q-Orbitrap system was connected to the UHPLC system *via* a heated electrospray ionization and controlled by Xcalibur 4.1 software that was used for data capture and analysis. The electrospray ionization source was operated and optimized in negative ionization mode. The optimized parameters of mass spectrometry were: capillary temperature: 320°C; sheath gas (N_2_) flow rate: 35 arbitrary units; auxiliary gas (N_2_) flow rate: 10 arbitrary units; sweep gas flow rate: 0 arbitrary units; spray voltage: 2.8 kV; S-lens RF level: 50 V; auxilliary gas heater temperature, 300°C; scan mode: full MS: scan range: 80–1200 m*/z*, the ions of five kinds of target flavonoids including quercetin ([M-H]^-^, *m/z* 301.03428), kaempferol ([M-H]^-^, *m/z* 285.03936), myricetin ([M-H]^-^, *m/z* 317.02919), apigenin ([M-H]^-^, *m/z* 269.04445), luteolin ([M-H]^-^, *m/z* 285.03936) were extracted for quantitative and qualitative analysis; maximum injection time (IT): 200 ms; scan resolution, 70,000 FWHM (m/z/s); automatic gain control (AGC) target: 1.0e^6^.

### PTGS2 Inhibition Assay

PTGS2 activity was tested using the PTGS2 Inhibitor Screening Kit (Lot. S0168, Beyotime, Shanghai, China) according to the manufacturer’s protocols. Briefly, recombinant human PTGS2 (hPTGS2) was mixed with the substrate and increasing concentrations of Myr or with Celecoxib (a selective PTGS2 inhibitor as a positive control) in 96-well plates under physiological conditions (pH 7.4 at 37°C). Following 10 min incubation at 37°C, the plates were read on a fluorescence microplate reader (TECAN Infinite M200 Pro, Tecan Group Ltd., MA, United States) using a setting of ex/em at 560 and 590 nm, respectively. The inhibition rate of PTGS2 activity was calculated following the manufacturer’s instruction.

### Cell Culture

Both mouse macrophage cell line (RAW264.7) and human hepatocyte cell line (L02) were purchased from the Cell Bank of Typical Culture Preservation Committee of Chinese Academy of Sciences (Shanghai, China). RAW264.7 cells were cultured with 10% FBS and DMEM in 5% CO_2_ at 37°C. L02 cells were cultured with 10% FBS, 1% insulin, 0.1% dexamethasone, and DMEM in 5% CO_2_ at 37°C.

### Establishment of NASH Model *In vitro* and Cell Treatment

RAW264.7 cells were incubated with 1 ng/ml LPS for 18 h to establish an inflammatory model of NASH *in vitro* (Liu et al., 2019). RAW264.7 cells were treated with 6.25, 25, and 100 μg/ml POL and 20 μM Myr for 18 h.

L02 cells were incubated with 1 mM free fatty acids (FFAs, oleic acid: palmitic acid = 2: 1) for 24 h to establish a lipid overloaded model of NASH *in vitro* (Li M et al., 2018). L02 cells were treated with 6.25, 25, and 100 μg/ml POL and 20 μM Myr for 24 h.

### Assessment of Cell Viability in L02 and RAW264.7 Cells With CCK-8

L02 and RAW264.7 cells were incubated with different concentrations of POL (0, 6.25, 12.5, 25.0, 50.0, and 100.0 μg/ml) for 24 h. Cytotoxicity was evaluated using a Cell Counting Kit-8 following the manufacturer’s protocol.

### ELISA of Inflammatory Cytokines in Supernatant

RAW264.7 cells were cultured and treated in six-well plates. The culture medium was collected and centrifuged at 2500 rpm and 4°C for 10 min. The supernatant was collected and tested with IL-6, TNF-α, and IL-1β ELISA kits in accordance with the manufacturer’s instruction.

### Immunofluorescence Assay

L02 cells were cultured and treated for 24 h in 48-well plates. The cells were fixed with 4% paraformaldehyde for 30 min and incubated with 0.05% Triton-X 100 for 15 min. After washing with PBS, the cells were incubated with 5% BSA in PBS buffer for 30 min and immersed in 2.5‰ PTGS2 primary antibody-5% BSA (v/v) for 12 h. The cells were washed again with PBS and incubated with FITC-conjugated secondary antibody for 1 h at 37°C. Images were observed and photographed by laser confocal microscopy (FV10C-W3, Olympus Corp., JPN).

### Oil Red O Stain Assay

L02 cells were cultured and treated in 24-well plates. The cells were fixed with 4% paraformaldehyde for 30 min and dyed with oil red O working solution for 15 min. The cells were stained with hematoxylin for 2 min to visualize the nuclei. The cells were observed and photographed by an inverted microscope.

### Determination of the TG Contents in L02 Cells

After washing with cold PBS, L02 cells were filled with 1 ml of PBS per 5–10×10^6^ cells and crushed with ultrasonic wave at 200 W for 3 s. The cell lysates were transferred to a 1.5 ml EP tube. TG content was measured using a TG test kit according to the manufacturer’s protocols.

### Real-Time Polymerase Chain Reaction

L02 cells were cultured and treated in 6-well plates. The cells were lysed, and total mRNA was extracted by Trizol method. Reverse transcription and PCR were conducted as previously described (Huang et al., 2019). The primers (Supplementary Table S1) were signed and synthesized by Takara Bio (Takara Biotechnology Inc., Dalian, CHN).

### Western Blotting

RAW264.7 cells or L02 cells were cultured and treated in 6 cm dishes. The cells were lysed, and total proteins were extracted by 2% phosphatase inhibitor and 2% protease inhibitor-RIPA lysis buffer (v/v). Western blot was operated by methods as previously described (Zhao et al., 2017). The primary and secondary antibodies (Supplementary Table S2) were purchased from Abcam (Abcam Inc., Shanghai, CHN).

### Statistical Analyses

Data were expressed as mean ± standard deviation. Statistical analyses were performed by one-way analysis of variance (ANOVA) and least-significant difference (LSD) test. Values at *p* < 0.05 were considered statistically significant.

## Results

### POL Alleviates Hepatic Steatosis and Inflammation in NASH Mouse Model Induced by MCD Diet

**FIGURE 1:**
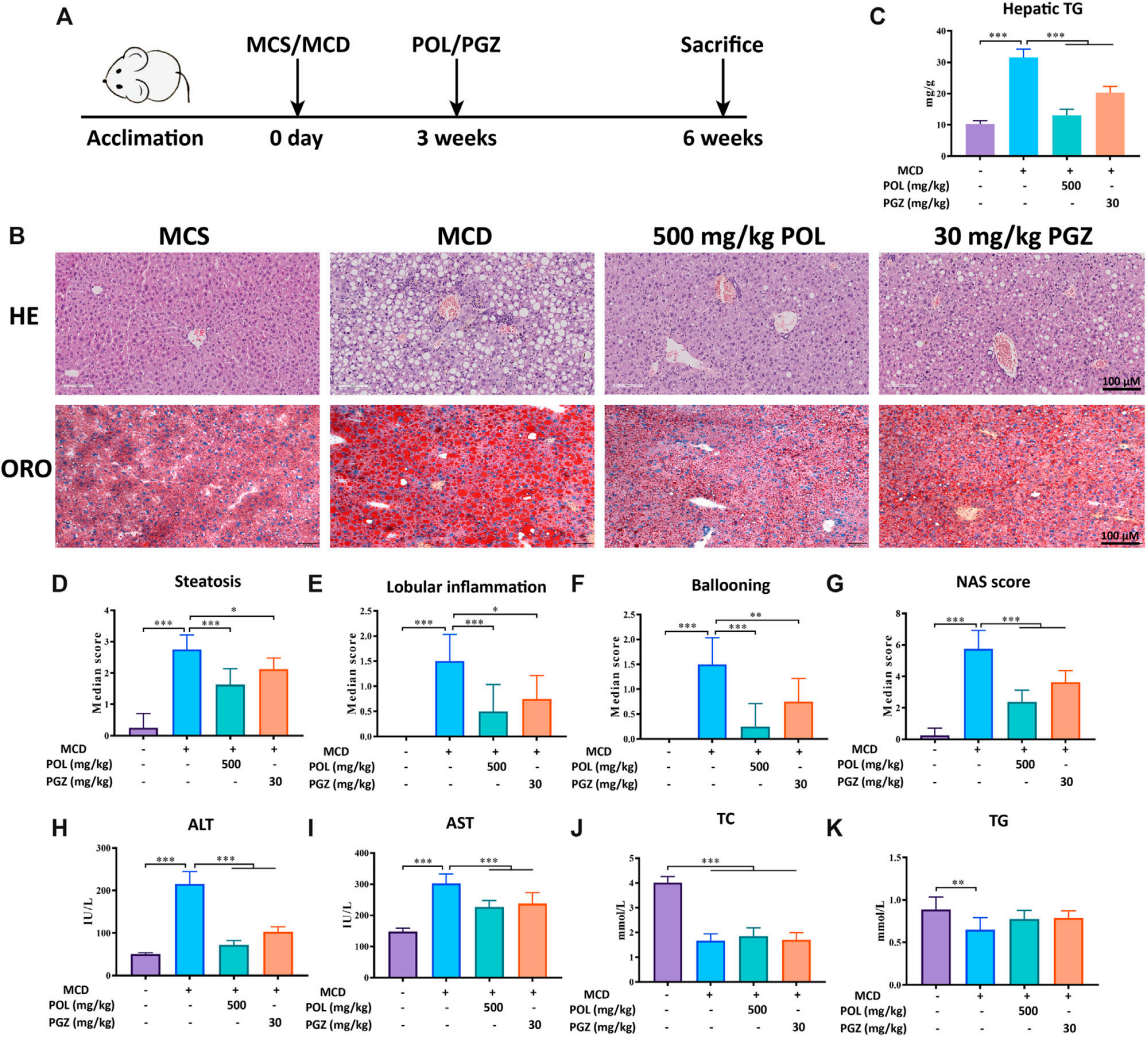
The effects of POL in the treatment of NASH in MCD diet-induced mouse model. Male C57BL/6 mice were fed with MCD diet for 6 weeks to establish a NASH model and treated with 500 mg/kg POL or 30 mg/kg PGZ for 3 weeks **(A)**. HE and oil red O staining (ORO) were performed to observe the histopathological characteristics of liver tissue after the treatment of POL **(B)**. Then, the NAS score **(G)**, including hepatic steatosis **(D)**, lobular inflammation **(E)**, and ballooning degeneration (**F**), were calculated. The specific classification criteria were as follows: 1) hepatic steatosis (<5%, 0 points; 5–33%, 1 point; 33–66%, 2 points; >66%, 3 points); 2) lobular inflammation (none, 0 points; < 2 lesions/200× view, 1 point; 2–4 lesions/200× view, 2 points; > 4 lesions/200× view, 3 points); and 3) ballooning degeneration (none, 0 points; rare, 1 point; extensive, 2 points). The contents of hepatic TG **(C)**, serum ALT **(H)**, AST **(I)**, TC **(J)**, and TG **(K)** were detected. Comparing with model group, *p < 0.05, **p < 0.01, ***p < 0.001.

C57BL/6 mice were fed with MCD diet for 6 weeks to induce NASH *in vivo* (Mridha et al., 2017) (Figure 1A). Prior to sacrifice, the mice were treated with 500 mg/kg POL extract for 3 weeks, while pioglitazone (PGZ) was used as a positive drug. Hepatic steatosis, lobular inflammation, and hepatocyte ballooning degeneration were observed in the liver tissues of model mice. After treated, POL and PGZ significantly decreased the degree of hepatic steatosis, inflammation, and ballooning comparing the model group (Figures 1B,D–G). And POL and PGZ prominently decreased the levels of hepatic TG (Figure 1C). The serum biochemical tests showed that POL and PGZ also significantly decreased the contents of AST and ALT comparing the model group (Figures 1H,I). But the contents of serum TG and TC have no significant differences between the model group, POL group, and PGZ group (Figures 1J,K). POL showed a remarkable therapeutic effect in the treatment of NASH.

### Active Components and Predicted Targets of POL for Treating NASH

**FIGURE 2:**
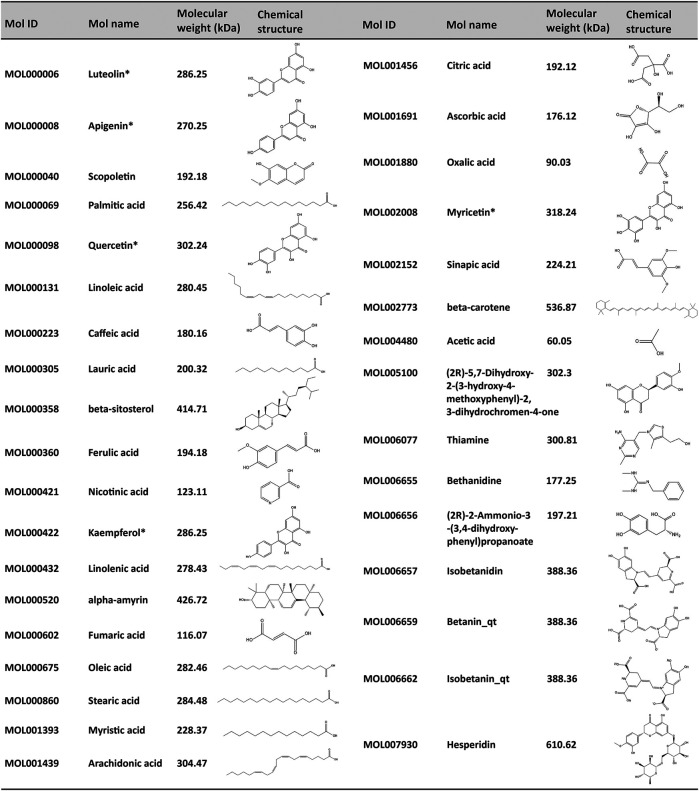
Corresponding compounds of N&P from POL in the treatment of NASH. Thirty four corresponding components of POL were closely related to the core genes (N&P) and obtained from TCMSP database. The Mol ID, Mol number, molecular weight, and structure of these compounds were listed. *Potential flavonoids from POL. “_qt” represented losing glucoside.

**FIGURE 3:**
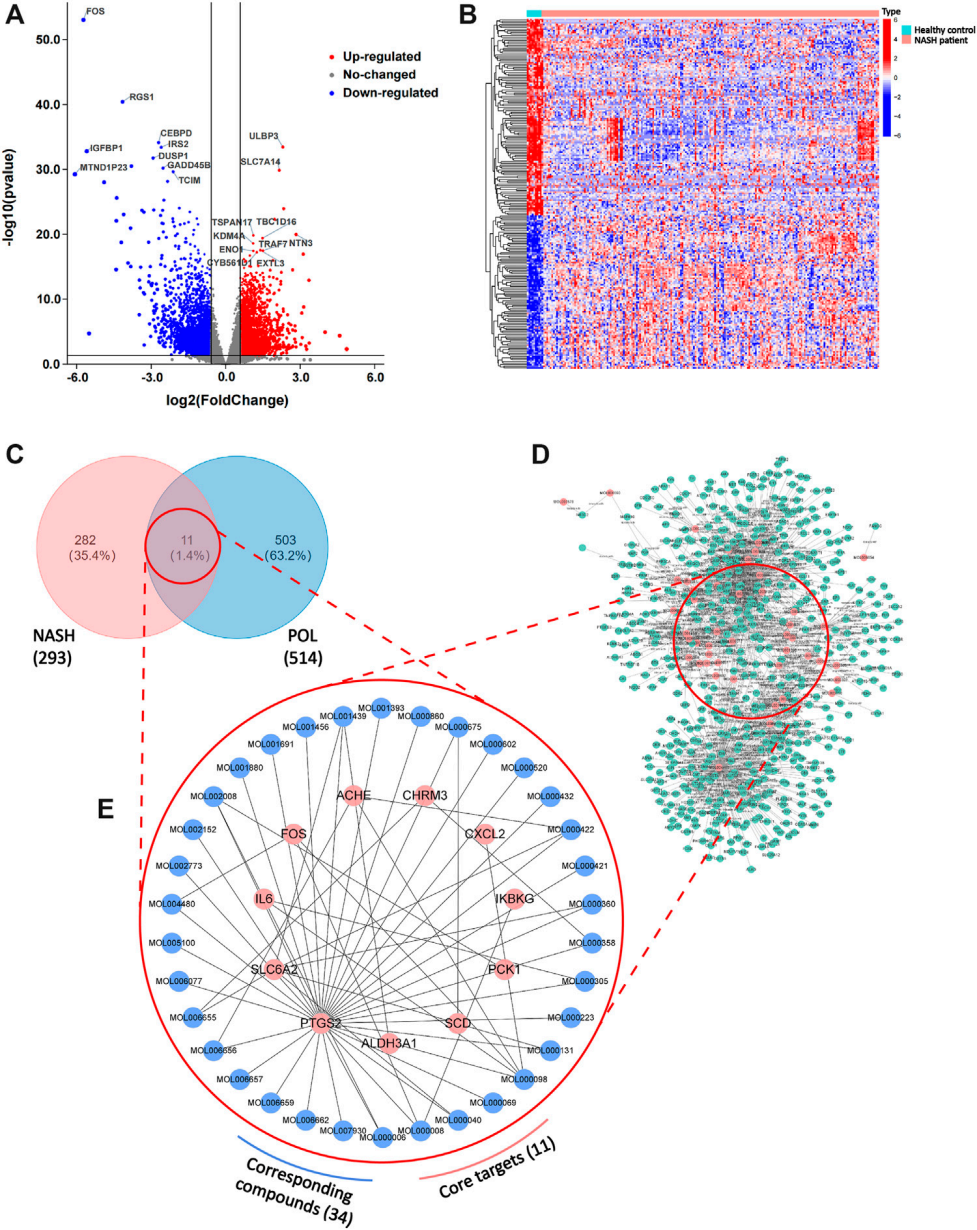
DEGs in patients with NASH and healthy people and the intersected genes of DEGs and predicted targets. The dataset (GSE135251) was downloaded from GEO Datasets. Then, DEGs were calculated by limma package and plotted by R. Volcano plot of DEGs **(A)** and heatmap plot of DEGs **(B)** were graphed and red dots represented up-regulated genes while blue represented down-regulated genes. Top ten up-regulated and down-regulated genes were labeled. Then, predicted targets of POL **(D)** were obtained from TCMSP database. The intersected genes (N&P) of DEGs and predicted targets **(C)** were graphed by Venn diagram. The network of corresponding compounds and core genes (N&P) **(E)** was graphed by Cytoscape. Blue circles represented constitutes of POL while red represented core genes (N&P).

To further elucidate the active components and mechanism of POL in treating NASH, total 44 active components and 514 predicted targets of POL were retrieved from TCMSP, BATMAN, and TCMID databases (Figure 3D). Then, we searched the high-throughput sequencing data of liver tissues (GSE135251) from GEO DataSets, and finally focused 293 differentially expressed genes (DEGs) between the patients with NASH and healthy people. The 514 predicted targets were intersected with 293 DEGs to gain 11 targets of POL in treating NASH (N&P: IL-6, FOS, SCD, CXCL2, PTGS2, IKBKG, PCK1, ALDH3A1, SLC6A2, ACHE, and CHRM3) (Figures 3C,E). Thirty-four components were closely related to these 11 targets of POL in treating NASH according to TCMSP database (Figure 2). The interaction network of active components and predicted targets was presented by a network interaction map (Figure 3E).

The cohort of GSE135251 contained a total of 216 frozen liver biopsies originated from 206 NASH cases and 10 healthy controls. DEGs were calculated by LIMMA R package with statistical power at |logFC| > 1.5 and adj.*p* < 0.05. The volcano plot shows the top 10 up-regulated and down-regulated DEGs (Figure 3A). The heatmap shows that the clustering of DEGs into 3 types, inflammatory factors, lipid metabolism, and production of extra-cellular matrix were the main fields (Figure 3B).

### Deep Bioinformatics Analysis to Screen the Core Targets of POL in Treating NASH

**FIGURE 4:**
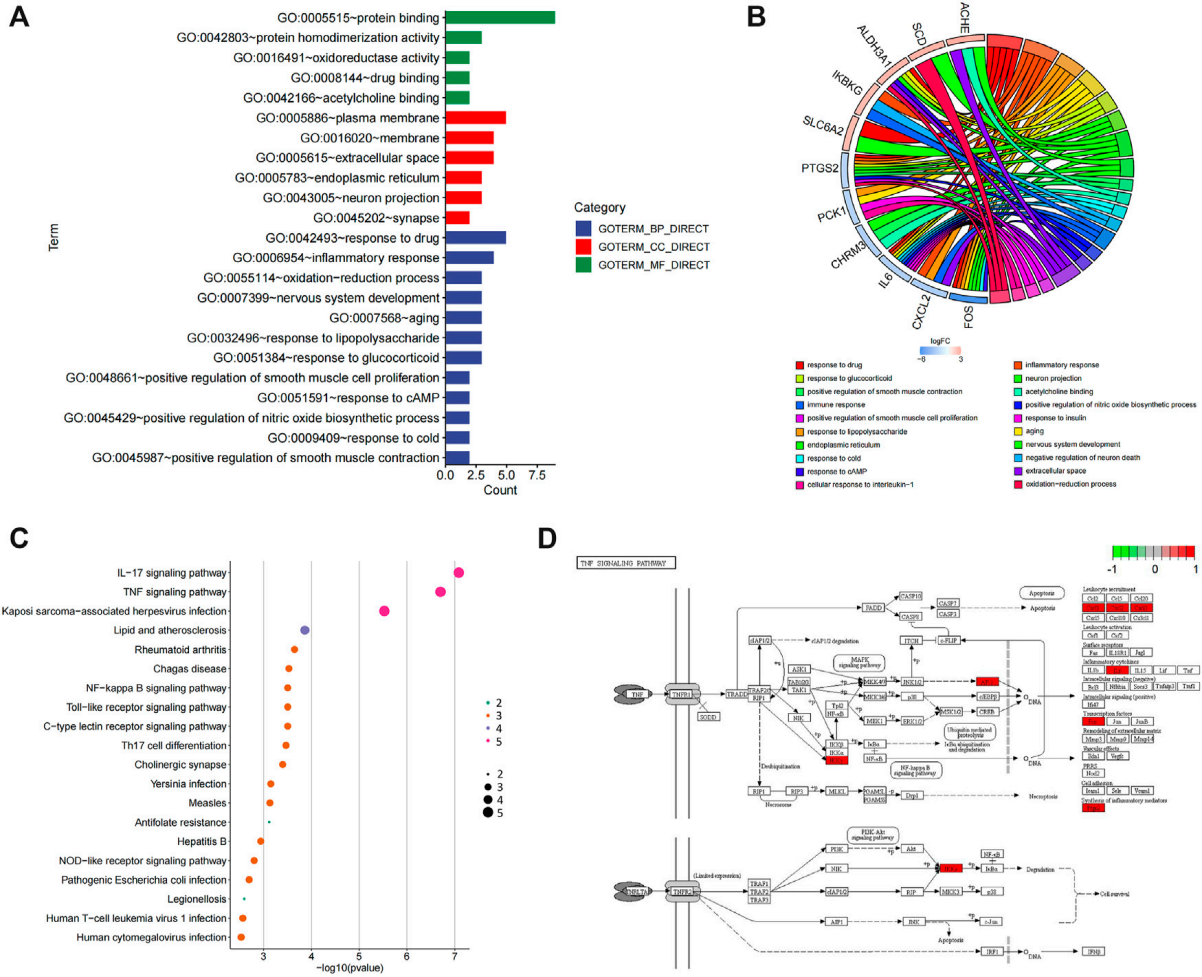
GO and KEGG enrichment of N&P. The core intersected genes (N&P) were analyzed by GO enrichment **(A,B)**. The green, red, and blue boxes represented biological process, cellular component, and molecular function, respectively. The top five results of GO enrichment were shown. Then, N&P were analyzed by KEGG enrichment **(C)**. The top twenty signaling pathways were listed and the pink circles represented the top three signaling pathways. The TNF signaling pathway, one of the top two signaling pathways, was analyzed **(D)**. The red boxes represented the core genes (N&P) in the TNF signaling pathway.

Bioinformatics analysis was employed to further analyze the core targets of POL in the treating NASH (Tian et al., 2019). To identify potential affected pathways of POL in treating NASH, KEGG pathway enrichment analysis was performed using the 11 targets from N&P. We found that “IL-17 signaling pathway” and “TNF signaling pathway” were significantly enriched (*p* < 0.05) (Figures 4B,C). According to the literature, IL-17 signaling accelerates the progression of NAFLD (Harley et al., 2014). IL-17 is also a risk factor for atherosclerosis in obesity-related NAFLD patients (Tarantino et al., 2014). And TNF signaling is closely related to the development from NAFL to NASH (Lu et al., 2021). To further identify the biological processes of the core genes (N&P), we did GO terms enrichment analysis and found the most significantly enriched terms are “inflammatory response” and “oxidative reduction process” (Figure 4A). These results indicate that POL may influence these signaling pathways and biological processes, thus decreasing inflammatory mediators and alleviating hepatic inflammation in NASH models.

**FIGURE 5:**
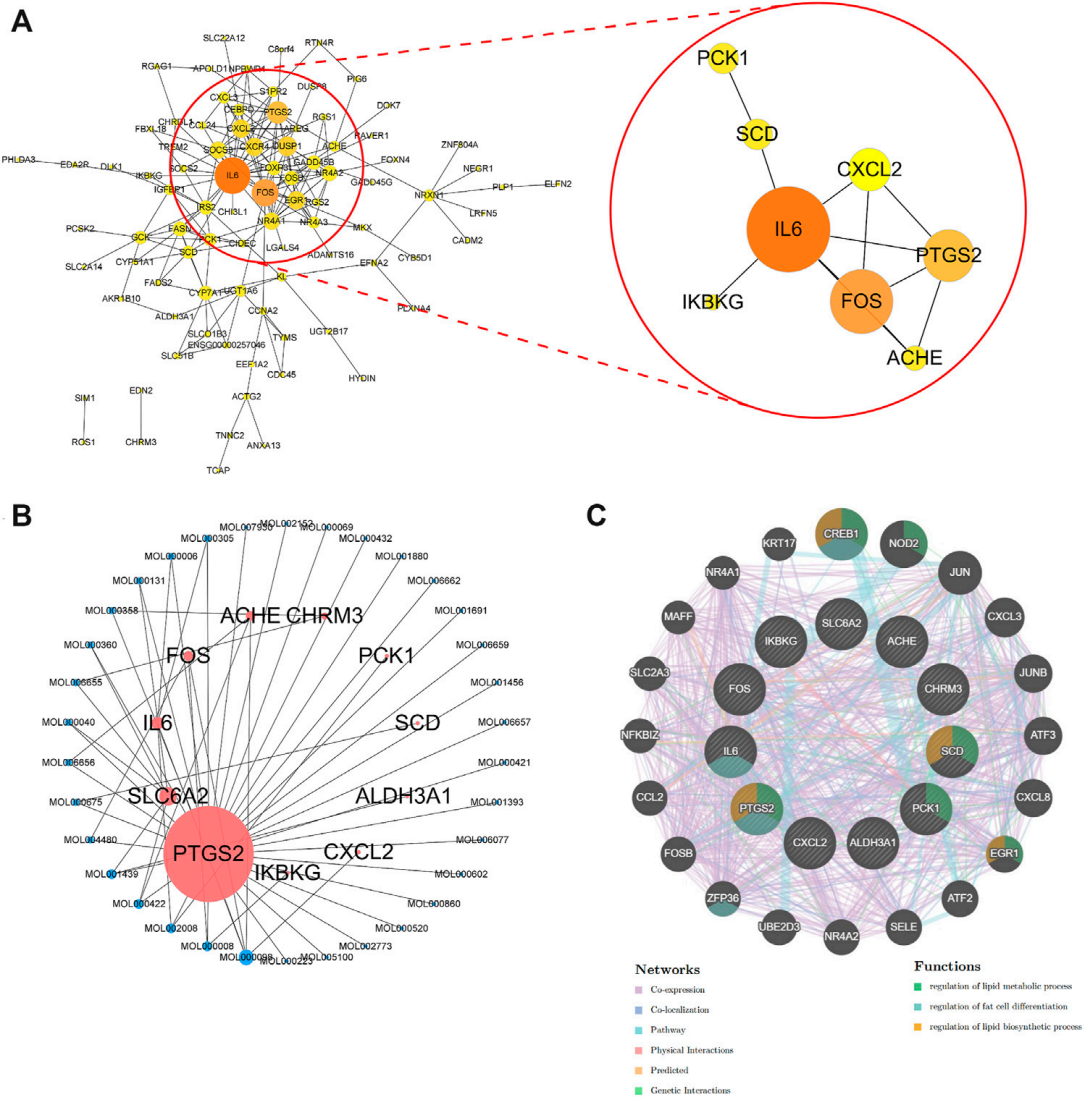
Protein-protein network analysis and topological analysis of N&P. Protein-protein network of core genes **(A)** was graphed by Cytoscape. The network of corresponding components from POL and core genes (N&P) **(B)** was graphed by Cytoscape. Pink node represented the core genes while blue represented the corresponding components. Node size is proportional to its degree. The larger the area is, the more important it is in the network. Topological network of N&P **(C)** was graphed by GeneMANIA. The different color lines represented the network relationships while the different color circles represented the gene functions.

To further identify the core target of POL in treating NASH, we did the topology analysis of protein–protein interaction and found that IL-6, FOS, PTGS2, and CXCL2 were the top 4 targets of N&P (Figure 5A). And the drug-target interaction network shows that PTGS2, SLC6A2, IL-6, and FOS were the core 4 targets of N&P (Figure 5B). On the other hand, we found that PTGS2 also participates in the synthesis of inflammatory mediators in the TNF signaling pathway which is the second significantly enriched pathway in KEGG analysis (Figure 4D). And PTGS2 is also the top gene participating the lipid metabolic process, fat cell differentiation, and lipid biosynthesis according to topological analysis of N&P (Figure 5C). These deep bioinformatics analyses suggest that PTGS2 may be the key target of POL in the treatment of NASH.

### Determination of the Contents of Five Flavonoids in POL and Identifying the Efficacious Flavonoids for Treating NASH

**FIGURE 6:**
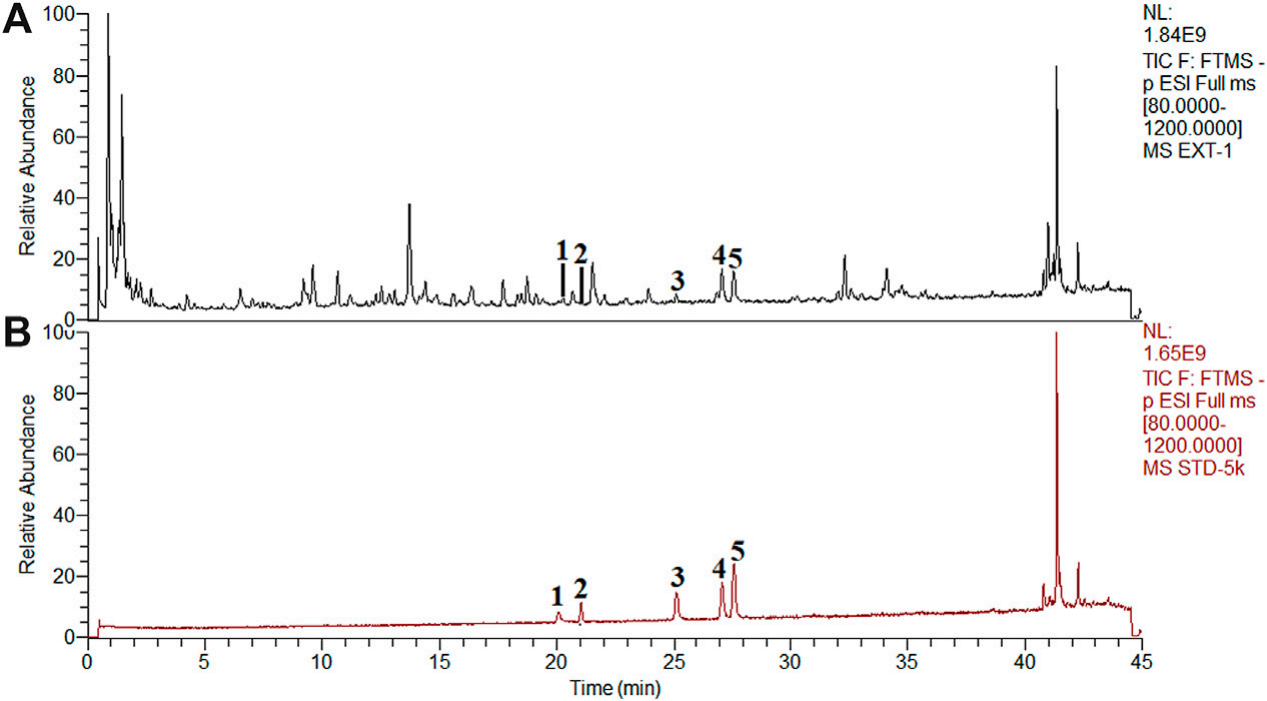
Chemical analysis of POL by UHPLC-Q-Orbitrap HRMS. The typical fngerprint chromatograms of the extract of POL **(A)** and reference standards **(B)**. (1) Myricetin. (2) Quercetin. (3) Luteolin. (4) Kaempferol. (5) Apigenin.

According to drug-target interaction network (Figure 5B), 5 flavonoids (myricetin, quercetin, luteolin, kaempferol, and apigenin) were the top five components closely related to PTGS2, IL-6, CXCL2, and FOS. So we detected the typical chromatographic fingerprints of the extract of POL and measured the contents of these five compounds (Figures 6A,B). The contents of myricetin, quercetin, luteolin, kaempferol, and apigenin in the extract of POL were 43.2 μg/g, 1.2 μg/g, 73.5 μg/g, 225.9 μg/g, and 138.0 μg/g, respectively. As the low level of quercetin, we only observed the rest flavonoids except quercetin in the further researches.

**FIGURE 7:**
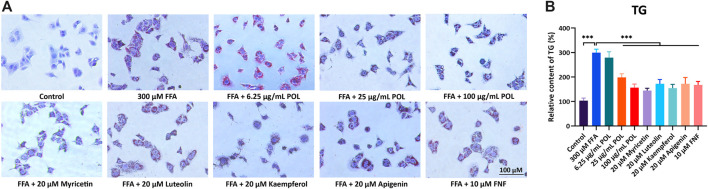
The comparison of anti-lipid accumulation in different concentration of POL and four flavonoids from POL. L02 cells were incubated with 300 μM FFA (oleic acid: palmitic acid = 2: 1) and treated with different concentrations of POL and 20 μM myricetin, luteolin, kaempferol, and apigenin for 24 h, respectively. Lipid accumulation and content of TG were evaluated by oil red O staining **(A)** and enzymatic colorimetric assay **(B)**. Comparing with model group, ***p < 0.001.

To compare the efficacy of 4 flavonoids from POL in treating NASH, we evaluated the effects of anti-steatosis by 4 flavonoids in human hepatocyte cells (L02). L02 was incubated with 300 μM FFAs for 24 h to establish NASH model *in vitro* (He et al., 2020) and treated with 20 μM myricetin, luteolin, kaempferol, apigenin, and 10 μM fenofibrate (FNF) for 24 h, respectively. Red oil O staining was operated to visualize the lipid droplets and the content of TG was detected. As results showed, all the 4 flavonoids reduced the lipids droplets and the levels of TG in L02, and Myr represented better effects than other flavonoids (Figures 7A,B). And we also evaluated the anti-inflammatory effects in LPS-induced RAW264.7 cells by those 4 flavnonoids. All the 4 flavonoids reduced the expressions of TNF-α and IL-6 in the supernatant significantly and Myr showed better effects among 4 flavonoids (Supplementary Figure 2A and B). These evidences suggest Myr may be the key active component of POL in the treatment of NASH.

### POL Down-Regulates PTGS2 and Regulates the Expression of *ACC1*, *CPT1a, SERBP1c*, *SCD1*, and *FASN* in L02 Cells

**FIGURE 8:**
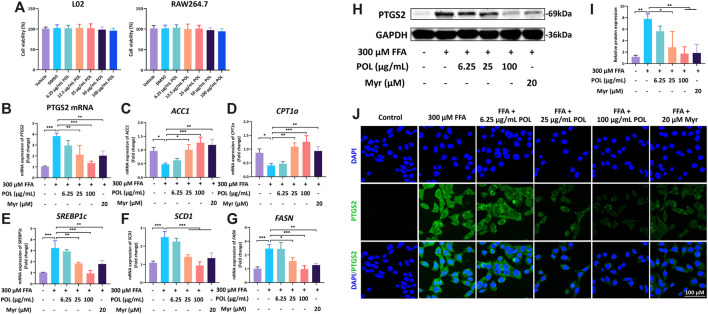
The effects of POL and Myr on the protein and mRNA expression of PTGS2 and the mRNA expression of *CPT1a*, *FASN*, *SCD1*, *ACC1*, and *SREBP1c* in FFA-induced L02. L02 cells were incubated with 300 μM FFA (oleic acid: palmitic acid = 2: 1) and treated with 6.25, 25, and 100 μg/ml POL and 20 μM Myr for 24 h, respectively. **(A)** Cell viabilities of L02 and RAW264.7 treated with 6.25, 25, and 100 μg/ml POL. The mRNA expression of *PTGS2* **(B)**, *ACC1* **(C)**, *CPT1a* **(D)**, *SREBP1c* **(E)**, *SCD1* **(F)**, and *FASN* **(G)** were determined by qt-PCR. Then, the protein expression of PTGS2 was detected by western blot **(H)** and immunoflurescence staining **(I)** and. The relative protein expression of PTGS2 was calculated **(J)**. Comparing with model group, *p < 0.05, **p < 0.01, ***p < 0.001.

Next, we employed validation experiments to further clarify these hypotheses. First, the viabilities of L02 and mouse macrophage cells (RAW264.7) were detected after the treatment with different concentrations of POL for 24 h by CCK-8. No cytotoxicity of POL was detected within 0–100 μg/ml in L02 and RAW264.7 cells (Figure 8A). We determined the expression of PTGS2 in L02 cells treated with different concentrations of POL or 20 μM Myr for 24 h. The mRNA and protein expressions of PTGS2 were evaluated by immunofluorescence, rt-PCR, and Western blot. After treatment, the mRNA and protein expressions of PTGS2 were significantly increased in FFA group, while these were significantly decreased in the medium- and high-dose POL groups and the Myr group (Figures 8B,H,I,J). And interestingly, the inhibition of PTGS2 in the Myr group is similar to the high-dose POL group. Because PTGS2 is an important enzyme in free fatty acids metabolism and a bridge linked with inflammation and lipid metabolism. The inhibitor of PTGS2 could significantly alleviate hepatic steatosis by regulating lipid synthesis (Wu et al., 2016). Then, we evaluated the mRNA expression of lipid synthesis and homeostasis signaling pathways. We found that the mRNA expressions of *ACC1* and *CPT1a* were significantly decreased in the FFA group, while those were significantly elevated in the medium- and high-dose POL groups and the Myr group (Figures 8C,D). And the mRNA levels of *SREBP1c*, *SCD1*, and *FASN* were significantly increased in the FFA group, while those were significantly decreased in the medium- and high-dose POL groups and the Myr group (Figures 8E–G). These findings clearly demonstrate that POL reduces the synthesis of lipids by inhibiting PTGS2 in hepatocytes and the main active component is Myr.

### POL Down-Regulates PTGS2 and Decreases the Secretion of IL-6, IL-1β, and TNF-α in RAW264.7 Cells

**FIGURE 9:**
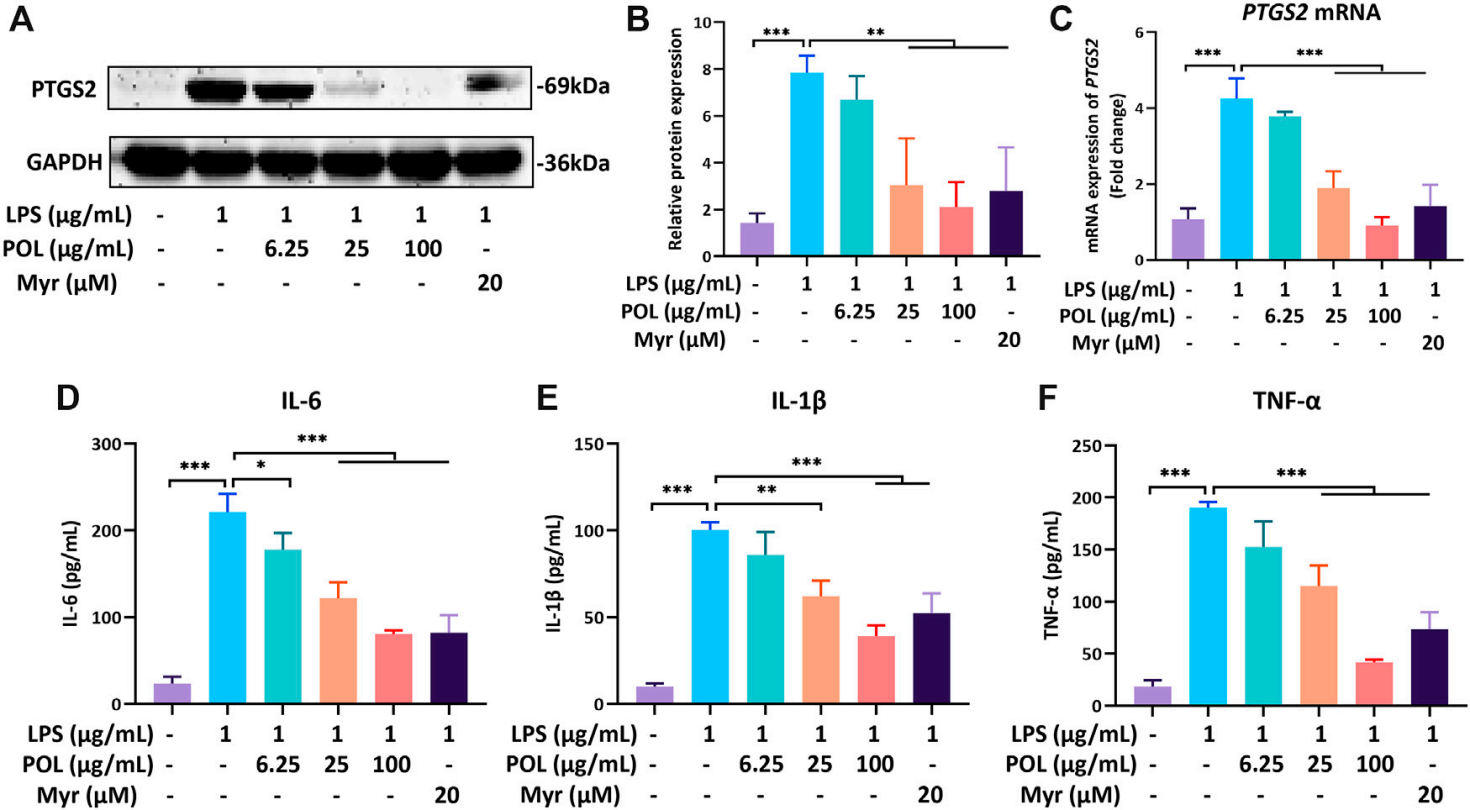
The effects of POL and Myr on protein and mRNA expression of PTGS2 and the secretion of IL-6, IL-1β, and TNF-α in LPS-induced RAW264.7. RAW264.7 was incubated with 1 μg/ml LPS and treated with 6.25, 25, and 100 μg/ml POL and 20 μM Myr for 18 h. Then, the protein expression **(A)** and mRNA expression of PTGS2 **(C)** were detected and the relative protein expression was calculated **(B)**. The cells were pretreated with 6.25, 25, and 100 μg/ml POL and 20 μM Myr for 1 h and exposed to 1 μg/ml LPS for 18 h. Then, the levels of IL-6 **(D)**, IL-1β **(E)**, and TNF-α **(F)** in culture supernatant were determined by ELISA. Comparing with model group, ^*^ *P* < 0.05, ^**^ *P* < 0.01, ***p < 0.001.

RAW264.7 was incubated with 1 μg/ml LPS for 18 h to simulate the hepatic inflammatory environment of NASH *in vitro* (Liu et al., 2019). After treatment with different concentrations of POL and Myr, RAW264.7 was collected to detect the mRNA and protein expressions of PTGS2. The results of rt-PCR and Western blot showed that the mRNA and protein expressions of PTGS2 were significantly increased in the FFA group, While these were significantly decreased in the medium- and high-dose POL groups and the Myr group (Figures 9A–C). The culture supernatant of RAW264.7 was also collected to detect the secretion of inflammatory mediators by ELISA. We found that the contents of IL-6, IL-1β, and TNF-α were increased significantly in the LPS group, while those were significantly decreased in the medium-dose and high-dose POL groups and the Myr group (Figures 9D–F). Myr also showed a similar inhibitory effect of PTGS2 and inflammation mediators to the high-dose POL in RAW264.7. These results suggest that POL inhibits the secretion of inflammatory mediators by down-regulating PTGS2 in macrophages and Myr is the key active component.

### PTGS2 Inhibition Activity of Myr

To verify whether Myr could directly inhibit the catalytic activity of PTGS2, we investigated the inhibitory effect of Myr against PTGS2 using a PTGS2 inhibitor screening kit *in vitro*. The hPTGS2 was co-incubated with increasing concentrations of Myr ranging from 0–100 μM. Then, the fluorescence signals were recorded and then the IC_50_ curves were plotted. Meanwhile, celecoxib (a known potent PTGS2 inhibitor) was used as a positive control. The results showed that Myr dose-dependently inhibited PTGS2, with the IC_50_ value of 16.30 μM (Supplementary Figure S1). These observations suggested that Myr is a moderate naturally occurring PTGS2 inhibitor. In brief, Myr could block PTGS2 related inflammatory responses *via* two ways, one is the down-regulation of PTGS2 in hepatocytes and RAW264.7 cells, and another one is directly inhibition on PTGS2.

## Discussion

The pathological mechanisms of NAFLD are quite complex. Dietary and exercise habits as well as environmental and genetic factors contribute to hepatic steatosis (Younossi et al., 2019). With further understanding of the relationship between NAFLD and obesity, diabetes, and other metabolic dysfunctional diseases, an increasing number of clinical doctors have reached an agreement on changing the name from NAFLD to metabolism-related fatty liver disease (MAFLD) (Eslam et al., 2020). Similar to the multiple systematic injuries of NAFLD outside the liver, chronic low-grade inflammation is not only observed in the liver but is also widespread in the adipose tissues, islets, and vascular endothelium within the spectrum of diseases, such as obesity, diabetes, and arteriosclerosis (Tilg et al., 2020). During the development from NAFL to NASH, lobular inflammation is the main characteristic that distinguishes simple hepatic steatosis and steatohepatitis. Inflammation is also one of the most central pathogenesis from the onset to evolution of NASH (Sutti and Albano, 2020). As initial pathological factors, dietary habits and environmental and genetic factors induce insulin resistance (IR), adipocyte proliferation, and changes in intestinal microbiome. IR promotes adipose tissue dysfunction with consequent altered production and secretion of adipokines and inflammatory cytokines (Khan et al., 2019). In another pathogenetic pathway, abnormal gut flora leads to intestinal barrier dysfunction, increased intestinal permeability, and thus increased circulating levels of molecules that contribute to the activation of inflammatory pathways and release of pro-inflammatory cytokines, such as interleukin 6 (IL-6) and tumor necrosis factor α (TNF-α). Inflammatory cytokines can activate immunocytes, such as macrophages, to maintain and enlarge inflammation, damage hepatocytes to induce liver injury, and activate hepatic stellate cells to deposit fibrogenetic matrix (Leung et al., 2016; Kazankov et al., 2019). Excessive lipid accumulation, insulin resistance, and leaky gut dysbiosis can induce the secretion of pro-inflammatory cytokines, trigger the polarize activation of macrophages, and irritate multiple inflammatory signaling pathways, which are the key pathological mechanisms in the change from NAFL to NASH and the so called “multiple-hit hypothesis” (Buzzetti et al., 2016).

POL is a common edible and medicinal plant widely distributed around the world. As an important vegetable in the Mediterranean diet, it presented a wide range of therapeutic effects, such as anti-inflammatory, anti-cardiovascular, and anti-cancer (Zhou et al., 2015). The leaves and seeds of POL could treat hyperlipidemia and hyperglycemia (Esmaillzadeh et al., 2015). A recent clinical study suggested that POL seeds have beneficial effects on fasting blood sugar, HOMA-IR, quantitative insulin sensitivity check index, TC, and LDL-c in patients with NAFLD (Gheflati et al., 2019). The seeds also have strong anti-oxidation and anti-inflammation effects (Baradaran et al., 2019). POL is a good source of omega-3 unsaturated fatty acid, which is effective in treating hyperlipidemia and NASH. POL is also rich in flavonoids, such as kaempferol, apigenin, myricetin, luteolin, and quercetin, which have a wide range of pharmacological effects (Miao et al., 2019; Melilli et al., 2020). However, the active components of POL and its pharmacological mechanism remain unclear. By using network pharmacology, we analyzed the differentially expressed genes (DEGs) between patients with NASH and healthy people, screened 293 genes, and finally focused on the core inflammatory cytokine PTGS2 through comparison with the pharmacological targets of POL.

Based on UHPLC-MS analysis, POL is rich in flavonoids such as myricetin, luteolin, kaempferol, apigenin, and quercetin. These flavonoids share a similar structure of two benzene rings joined by a three-carbon bridge (C6–C3–C6) and comprise diverse subclasses, which are called flavonols (quercetin and kaempferol) and flavones (myricetin, luteolin, and apigenin) (Peluso et al., 2015). Flavonoids exhibit immune regulatory effects. Clinical studies showed that the intake of flavonoid-rich vegetables or pure flavonoid (quercetin) could reduce the circulating levels of IL-6 and TNF-α (Ilaria et al., 2013). Flavonols, also named polyphenols, such as quercetin, can regulate the Th1/Th2 balance, inhibit the secretion of pro-inflammatory cytokines, and decrease leukotriene secretion (Jafarinia et al., 2020). In the present study, we predicted the inhibitory effects of five flavonoids from POL to PTGS2 through network pharmacological analysis. Then, we compared the efficacy of four flavonoids from POL extract in treating FFA-induced hepatocytes. Myricetin (Myr) performed the better effect of alleviating hepatic steatosis *in vitro*. According to recent studies, Myr could improve hepatic steatosis and liver inflammation by elevating Nrf2 signaling pathway in NAFLD mice (Xia et al., 2016). It also decreased the lipid accumulation in liver and serum by regulating PPARα in high-fat-diet induced rats (Chang et al., 2012). Microarray analysis of hepatic gene expression profiles showed that Myr significantly altered the expression profiles of 177 genes which were involved in 12 biological pathways, including the PPAR signaling pathway (Xia et al., 2016). We also investigated these targets regulated by myricetin *in vitro* and found it could also up-regulate the expressions of Nrf2 and PPARγ (Supplementary Figures S3A,B). So PTGS2 may be not the only one target of Myr. In this study, Nrf2 and PPARγ were also suggested as different expression genes between NASH patients and healthy people (*p* < 0.05; |logFC| = 0.38 and 0.31). As recent studies have reported these targets of myricetin in the treatment of NASH, we considered with the results suggested by network pharmacological analysis in this study, and finally focused on PTGS2 in the experimental researches.

**FIGURE 10:**
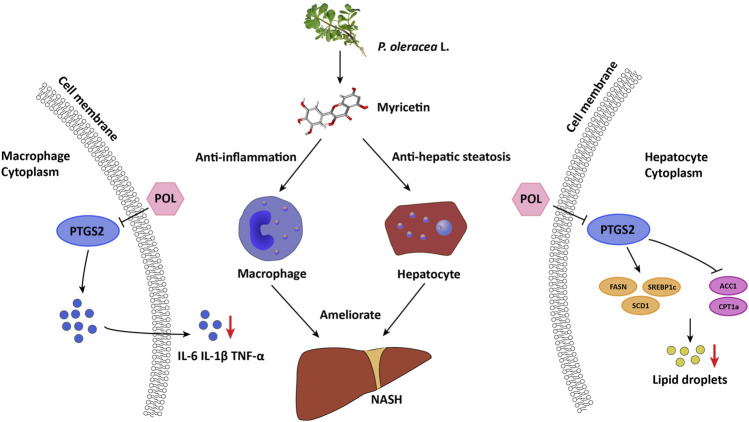
Proposed mechanism of POL and its main active component myricetin in treating NASH through inhibiting PTGS2. In macrophages, POL and myricetin could decrease the secretion of inflammatory mediators such as IL-6, IL-1β, and TNF-α through down-regulating PTGS2. In hepatocytes, POL and myricetin could regulate lipid synthesis and homostasis by down-regulating FASN, SREBP1c, and SCD1 and up-regulating ACC1 and CPT1a through inhibiting PTGS2. Thus, POL could reduce lipid accumulation of hepatocyte and inhibit hepatic inflammation to treat NASH.

PTGS2, also known as cyclooxygenase-2 or COX-2, is a key rate-limiting enzyme for synthesis of prostaglandin from arachidonic acid. Superoxides produced by PTGS2 can react with NO to trigger an oxidative cascade (Ferrer et al., 2019). During acute or chronic hepatic inflammation, increasing levels of PTGS2 in macrophages may contribute to liver injury through excessive generation of reactive oxygen species (ROS) and production of toxic prostanoids (Tsukayama et al., 2021). Several studies suggested that PTGS2 participates in the development of NASH. PTGS2 is altered in human and animal models with NASH. PTGS2 and its downstream prostaglandin E2 can interplay with Toll-like receptor/MyD88 signaling pathway to induce inflammation. PTGS2 also comprehensively participates in inflammatory signal transduction, such as in TNF and NF-κB signaling pathways (Echizen et al., 2016). Selective PTGS2 inhibitors could significantly ameliorate hepatic steatosis, inflammation, and liver injury in NASH models (Wu et al., 2016). In our *in vitro* experiments, medium- and high-dose POL and Myr significantly decreased the expression of PTGS2 and inhibited the secretion of IL-6, IL-1β, and TNF-α in RAW264.7 cells induced by LPS. These results suggest that POL and Myr could exert anti-inflammatory function by regulating PTGS2 (Figure 10).

As a bridge of inflammation and oxidative stress, PTGS2 is not only an inflammatory cytokine producer but also an important enzyme in lipid metabolism (Clemente et al., 2020). As the products of PTGS2, prostanoids are a superfamily of lipid mediators synthesized from arachidonic acid. COX-2 could metabolize neutral lipids, including endocannabinoid-like esters and amides. Knock out of COX-2 could inhibit lipid accumulation in HepG2 cells by promoting mitophagy (Chen et al., 2019; de Bus et al., 2019). Cox-2 could also affect the lipid signal transduction by regulating fatty acid synthesis enzyme such as FASN (Qiu et al., 2019). In the present study, we found that medium- and high-dose POL and Myr significantly decreased the number of lipid droplets in hepatocytes induced by FFA, up-regulated the expression of *ACC1* and *CPT1a*, and down-regulated the expression of *SREBP1c*, *SCD1*, and *FASN*, which are closely related to the synthesis and homeostasis of lipids. Moreover, medium- and high-dose POL and Myr significantly decreased the expression of PTGS2 in hepatocytes, similar to that in macrophages. And we also found that POL can reduce the expression of hepatic PTGS2 in the murine model with NASH (Supplementary Figure 4). These results suggest that POL and Myr may inhibit the lipid synthesis to alleviate NASH by regulating PTGS2 and its downstream lipid signaling pathways (Figure 10).

Our work showed that high-dose POL showed better effects than Myr on anti-inflammation and anti-hepatic steatosis. In addition to flavonoids, POL is rich in unsaturated fatty acids, such as α-linolenic acid and linolenic acid, alkaloids, ascorbic acid, and carotenoids (Zhou et al., 2015). These components also exhibit biomedical activities in treating NASH. Omega-3 and omega-6 fatty acids, which are polyunsaturated fatty acids (PUFA), can reduce lipid accumulation, ameliorate oxidative stress, and inhibit lipid peroxidation (Li T. T et al., 2018). Carotenoids, which are called pro-vitamin A, can be transformed into vitamin A in the liver and functions against anti-oxidative stress, which is closely related to obesity, diabetes, and arteriosclerosis (Saeed et al., 2017). These active compounds are warranted to be further studied to evaluate the efficacy and elucidate the underlying mechanism of POL for treatment of NASH.

## Conclusion

This study elucidated the possible mechanism and core active components of POL and evaluated its efficacy in treating NASH by integrating bioinformatic analysis and validation experimental pharmacology. *In vivo*, POL has a prominently effect of alleviating hepatic steatosis and liver injury. Then, as the bioinformatics analysis suggests, PTGS2 is the core target and Myr is the key constituent in POL for treating NASH. *In vitro*, POL and Myr can significantly down-regulate the expression of PTGS2, decrease the number of lipid droplets, and regulate the mRNA expression of lipid synthesis and homeostasis genes, including *FASN*, *CPT1a*, *SERBP1c*, *ACC1*, and *SCD1* in hepatocytes. And POL and Myr can also significantly reduce the expression of PTGS2 and block the secretion of inflammatory mediators TNF-α, IL-6, and IL-1β in macrophages. Further investigations demonstrate that Myr acts as both suppressor and inhibitor of PTGS2. Collectively, POL and its major component Myr can ameliorate NASH *via* down-regulating and inhibiting PTGS2, suggesting that POL and Myr can be developed as novel medicines for treating NASH.

## Data Availability

The datasets presented in this study can be found in online repositories. The names of the repository/repositories and accession number(s) can be found in the article/Supplementary Material.

## Abbreviations

BATMAN, bio-informatics analysis tool for molecular mechanism; DEGs, differentially expressed genes; FFA, free fatty acids; GO, gene ontology; HPLC, high performance liquid chromatography; IKBKG, inhibitor of nuclear factor κ B kinase regulatory subunit γ; IL-6, interleukin-6; CXCL2, chemokine (C-X-C motif) ligand 2; KEGG, kyoto encyclopedia of genes and genomes; LPS, lipopolysaccharide; MCD, methionine- and choline-deficient diet; Myr, myricetin; NASH, non-alcoholic steatohepatitis; POL, *Portulaca oleracea* L.; PTGS2, prostaglandin- endoperoxide synthase 2; TCMID, traditional chinese medicine integrative database; TCMSP, traditional chinese medicine systems pharmacology database and analysis platform.
