# Supplementary figures and images for: Deciphering the Effective Constituents and Mechanisms of Portulaca oleracea L. for Treating NASH via Integrating Bioinformatics Analysis and Experimental Pharmacology
Source: Front Pharmacol. 2022 Jan 19;12:818227. doi: 10.3389/fphar.2021.818227 (PMC8807659; doi:10.3389/fphar.2021.818227)

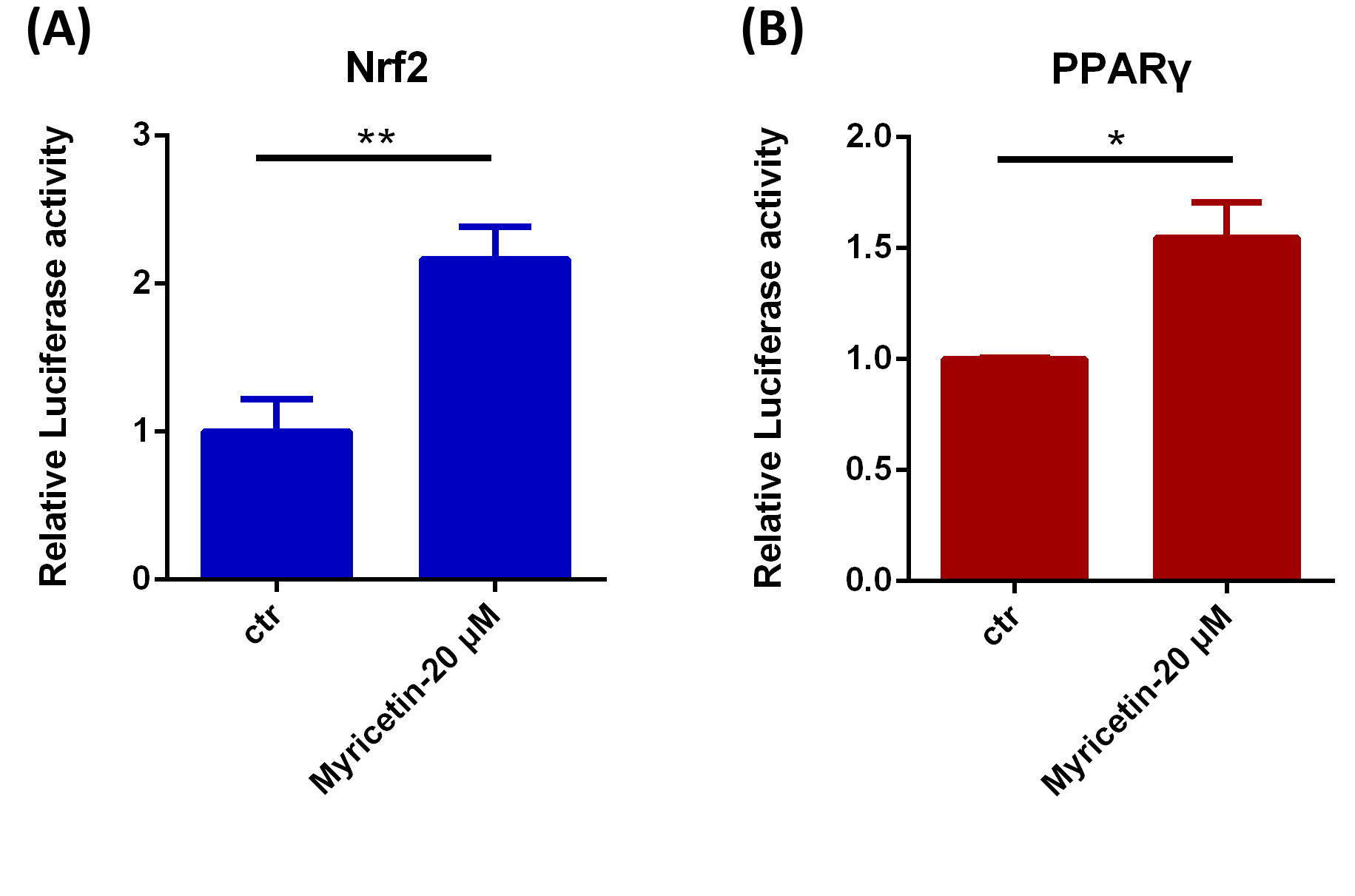

Supplement: Supplementary file 1 [file Image3.JPEG]

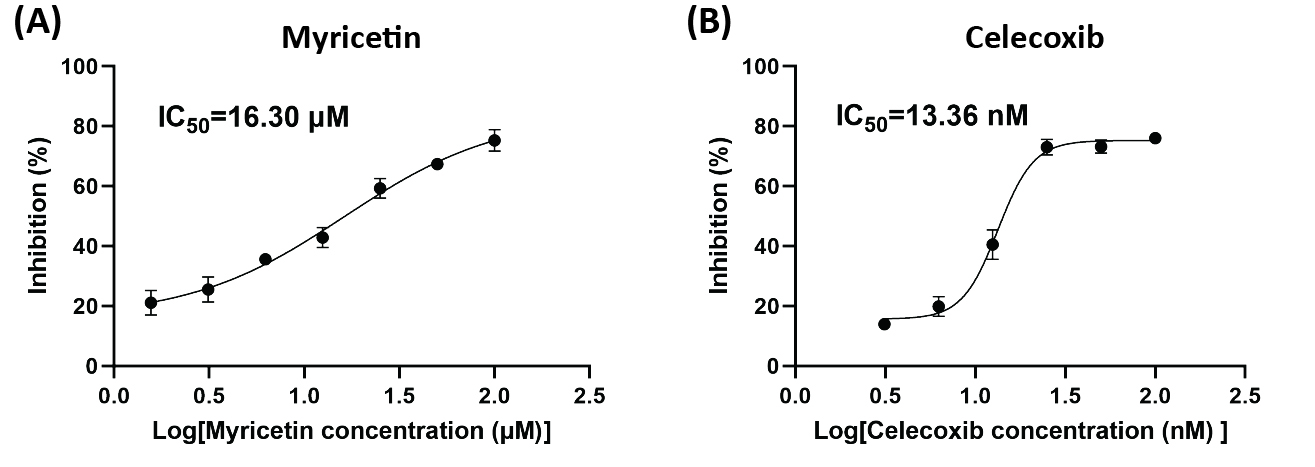

Supplement: Supplementary file 2 [file Image1.JPEG]

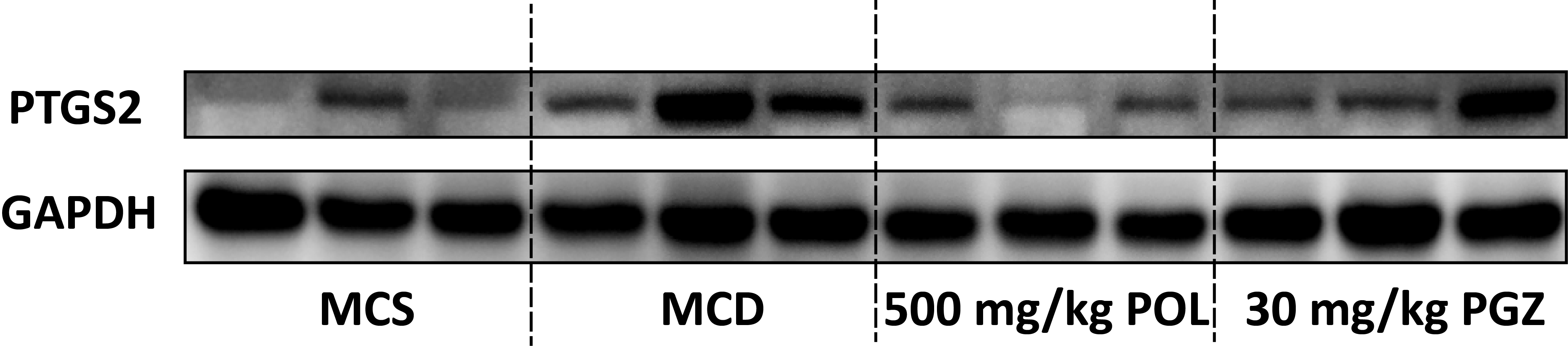

Supplement: Supplementary file 3 [file Image4.JPEG]

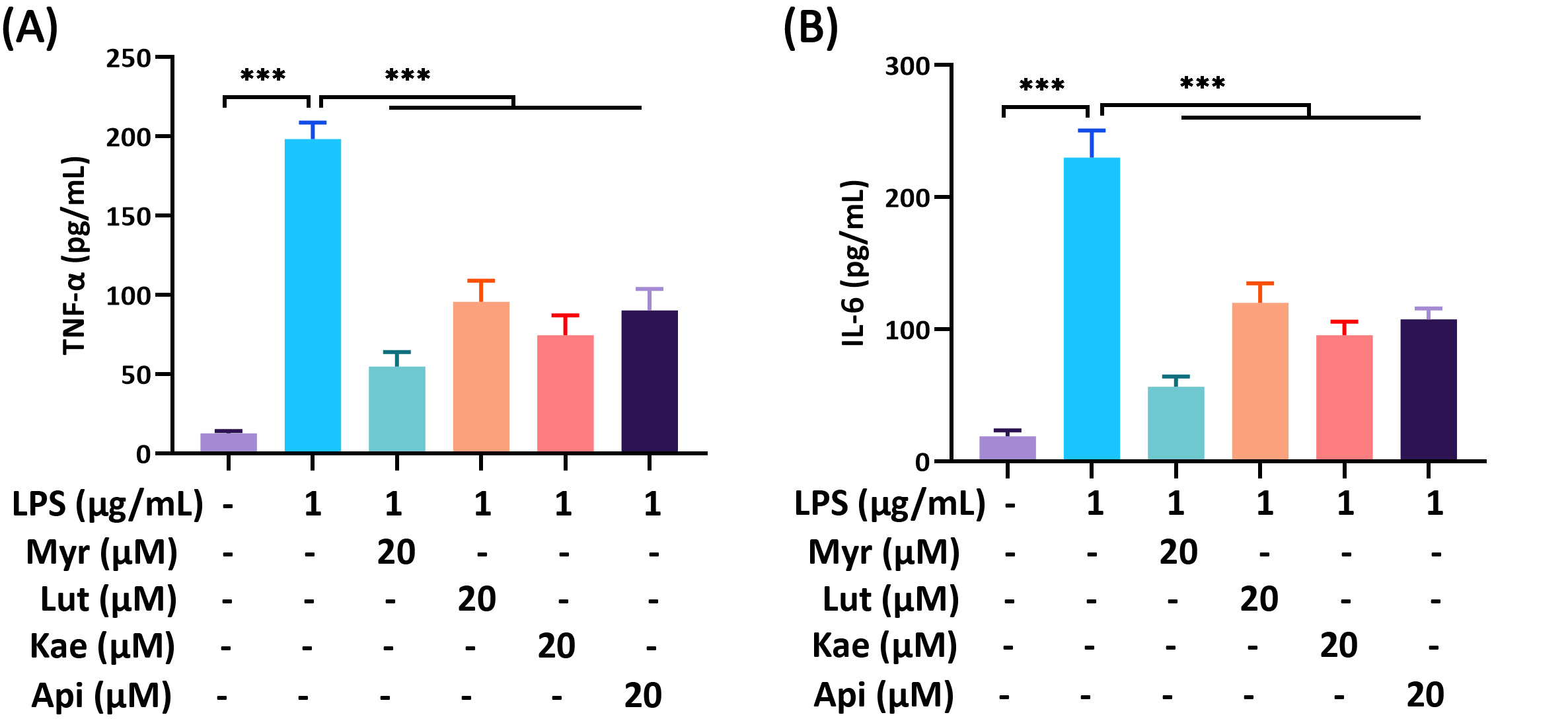

Supplement: Supplementary file 4 [file Image2.JPEG]
