# Supplementary material for: Deciphering the Effective Constituents and Mechanisms of Portulaca oleracea L. for Treating NASH via Integrating Bioinformatics Analysis and Experimental Pharmacology
Source: Front Pharmacol. 2022 Jan 19;12:818227. doi: 10.3389/fphar.2021.818227 (PMC8807659; doi:10.3389/fphar.2021.818227)
Supplement: Supplementary file 6 [file Table2.DOC]

**Supplementary Materials**

**Deciphering the effective constituents and mechanisms of *Portulaca* *oleracea* L. for treating NASH *via* integrating bioinformatics analysis and experimental pharmacology**

**Xiaoli He^1†^, Yiren Hu^1†^, Wei Liu^3^, Guanghao Zhu^2^, Ruoxi Zhang^1^, Jiawen You^1^, Yanting Shao^1^, Yunhao Li^1^, Zeng Zhang^1^, Jingang Cui^1^, Yanming He^1*^, Guangbo Ge^2*^, and Hongjie Yang^1*^**

^1^Department of Endocrinology, Research Laboratory of Pharmacy, Center of Experimental Animals, Clinical Research Institute of Integrative Medicine, Yueyang Hospital of Integrated Traditional Chinese and Western Medicine, Shanghai University of Traditional Chinese Medicine, Shanghai 200437, China;

^2^Shanghai Frontiers Science Center of TCM Chemical Biology; Institute of Interdisciplinary Integrative Medicine Research, Shanghai University of Traditional Chinese Medicine, Shanghai, 201203, China;

^3^Key Laboratory of Liver and Kidney Diseases (Ministry of Education); Institute of Liver Diseases, Shuguang Hospital Affiliated to Shanghai University of Traditional Chinese Medicine, 528 Zhangheng Road, Shanghai, 201203, China

***Correspondence:**

E-mail: heyanming176@163.com (Yanming He); geguangbo@dicp.ac.cn (Guangbo Ge); yyyanghongjie@163.com (Hongjie Yang)

^†^These authors have contributed equally to this work and share first authorship.

**Supplementary Table 2**

|  | Antibodies | Anti-species | Inc. | Dilution | Lot. |
| --- | --- | --- | --- | --- | --- |
| Primary antibodies | PTGS2 | Rabbit | Abcam | 1: 1000 | ab179800 |
|  | GAPDH | Mouse | Proteintech | 1: 5000 | 60004-1-Ig |
| Secondary antibodies | Anti-mouse | Goat | Beyotime | 1: 1000 | A0216 |
|  | Anti-rabbit | Goat | Beyotime | 1: 1000 | A0208 |
|  | FITC-anti-rabbit | Goat | Abcam | 1: 3000 | ab6717 |

**Table S2.** The list of primary antibodies and secondary antibodies.
